# Decompressive hemicraniectomy for malignant middle cerebral artery infarction: Experience from the Western Province of Saudi Arabia

**DOI:** 10.17712/nsj.2017.3.20170051

**Published:** 2017-07

**Authors:** Haifa M. Algethamy, Afnan Samman, Saleh S. Baeesa, Mohammed A. Almekhlafi, Yousef A. Al Said, Ahmed Hassan

**Affiliations:** *From the Department of Critical Care Medicine (Algethamy), Division of Neurosurgery (Samman, Baeesa), Department of Internal Medicine (Almekhlafi), Faculty of Medicine, King Abdulaziz University, and from the Department of Neurosciences (Al Said, Hassan), King Faisal Specialist and Research Center, Jeddah, Kingdom of Saudi Arabia*

## Abstract

**Objective:**

To describe our experience implementing decompressive hemicraniectomy (DH) for eligible patients with malignant middle cerebral artery (MCA) infarcts.

**Methods:**

We retrospectively collected data of malignant MCA infarction patients requiring DH at King Abdulaziz University Hospital & King Faisal Specialist Hospital & Research Center, Jeddah, Kingdom of Saudi Arabia between October 2010 and July 2015. Clinical outcome was assessed immediately postoperatively using Glasgow Coma Score (GCS), and at 12 months using the modified Rankin scale (mRS) and Barthel index. Survival was evaluated at thirty-days and one year after surgery.

**Results:**

Six out of 10 patients diagnosed with malignant MCA infarction underwent DH. Among the surgically treated patients (n=6), 4 were males (66%), and the median age was 22.5 years. The median time from admission to surgery was 35.5 hours. The median post-operative GCS was 6.5. Three patients (50%) died within 30 days of DH. In those who survived, the median mRS was 4.5 and BI was 7.5.

**Conclusion:**

Decompressive hemicraniectomy saves life and has the potential of improving survival functional outcome when done fast and in carefully selected patients. We call for national awareness of the management of such cases and early intervention.

Despite recent advances in the management of acute stroke over the past couple of decades, the rates of mortality and morbidity remain high in those who suffer a hemispheric ischemic injury in the distribution of the middle cerebral artery (MCA).^1^-^5^ The high rates of death and poor neurological outcomes observed in the so-called ‘malignant stroke’ stem from the low rates of reperfusion of these major occlusions spontaneously or using intravenous thrombolysis. Therefore, widespread cerebral edema results from the large infarct size with a significant increase in the intracranial pressure (ICP), often leading to brain herniation.^6^-^11^ Malignant MCA infarction is characterized by early signs of neurological deterioration, raised ICP, as well as imaging evidence of mass effect and midline shift.^12^ Several randomized clinical trials have indicated a significant reduction in mortality and some measures of morbidity in patients treated with decompressive hemicraniectomy versus those treated conservatively.^13^-^15^ Despite these results, controversy exists regarding the patients’ selection criteria as well as the timing of surgery.^10^,^16^-^18^ This lack of consensus led to the variable application of the available evidence with a very wide range of performance of decompressive hemicraniectomy in clinical practice. The current report describes our local experiences treating malignant MCA strokes at 2 tertiary care centers in Jeddah, Saudi Arabia.

## Methods

We retrospectively reviewed the records of patients admitted to King Abdulaziz University Hospital and King Faisal Specialist Hospital & Research Center in Jeddah, Kingdom of Saudi Arabia, between October 2010 and July 2015, with the diagnosis of large hemispheric brain infarcts complicated by malignant edema and were referred to the neurosurgical service for consideration of surgical decompression.

### Inclusion/Exclusion criteria

We included all those who underwent decompressive hemicraniectomy with duraplasty for malignant MCA infarction. Indications for surgery were deterioration of the Glasgow Coma Score (GCS), despite adequate medical therapy, caused by the local brain edema, with midline shift or obliteration of basal cisterns.

### Surgical technique

**Figure 1:**
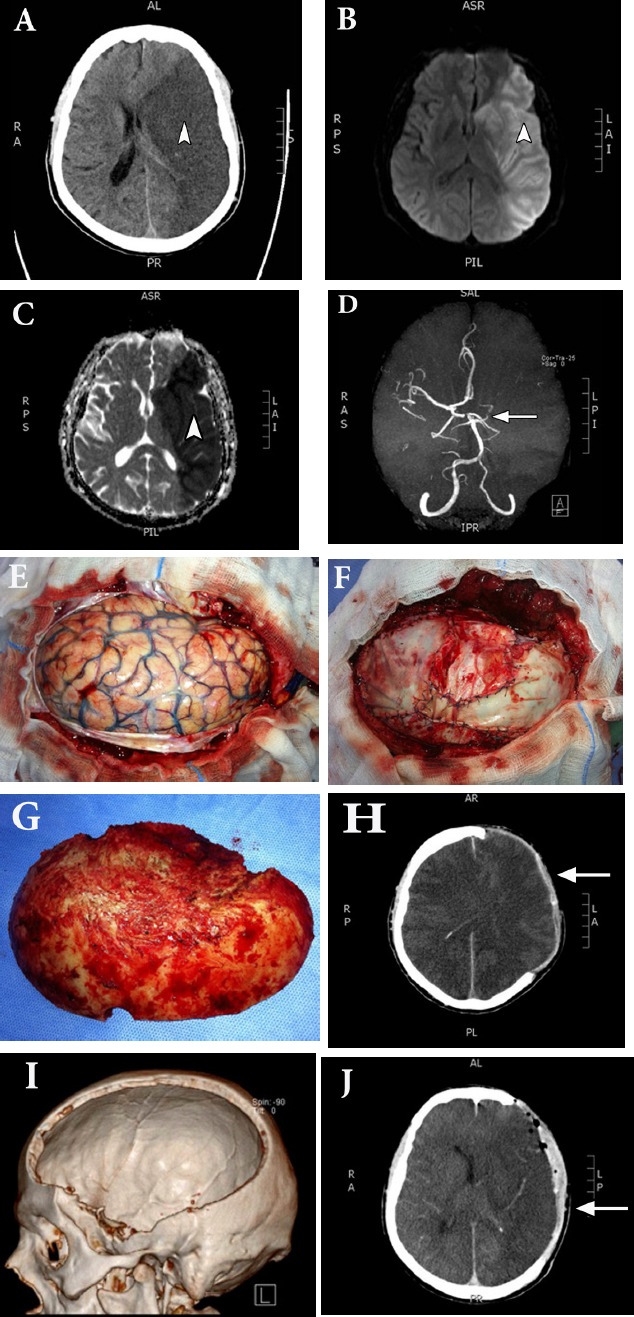
Case illustration of a 39-year-old male (case 4) who presented with left massive MCA infarction demonstrated on **A)** plain CT scan which was delineated by MRI diffusion **B)** and ADC scan **C)** due to occlusion of MCA (arrowhead) **D)**. He underwent left decompressive hemicraniectomy **E)** and duraplasty (F). G) The bone flap was 17.6 x 14.3 cm in maximum diameter. H) Postoperative CT scan showing marked swelling of the infarcted tissue and (I) adequate bone decompression (arrow). The swelling has subsequently decreased in size over 2 weeks (arrow) (J).

The frontotemporoparietal question mark skin incision, with a large flap base, is performed and sparing the superficial temporal artery and the temporal branch of the facial nerve. After elevating the skin flap with the attached temporalis muscle, an enormous bone flap (frontoparietoccipital length=14-18 cm) is created with the high-speed craniotome via 4-5 burr holes. The bone flap extends as far as the skull base in the temporal bone. The dura mater is then widely opened, either using a curved crescent-shaped or star-shaped incision. No resection performed for the infarcted brain tissue. A duraplasty is performed by using treated bovine pericardium patch and sutured in a water-tight fashion, and subcutaneous, and skin sutures are then performed (**Figure 1**). When considered necessary, a closed suction drain is sometimes placed subcutaneously for 24 hours. The bone flap is stored in the operating room refrigerator in a sterile sealed bag for the identified patient under -20 C temperature. Once the brain swelling subsides and confirmed with a CT scan, the bone flap is washed with betadine solution and saline, and then fixed, using microplates or craniofix skull clamps, via opening the same incision. There were no complications related to the cranioplasty procedure.

### Outcome

Functional outcome was measured at a 12-month follow-up and to with the modified Rankin Scale (mRS; 0=complete recovery, 6=death), and Barthel index (0-100). Mortality was assessed at 1 and 12 months after the surgery.

### Statistical analysis

Descriptive statistics were performed for the variables and expressed as frequency (percentage) and as mean (standard deviation, SD). Significance was set at *p*<0.05.

## Results

Ten patients were identified, among those, 6 patients underwent decompressive hemicraniectomy. **Table 1** shows the characteristics of those patients. Two patients were not offered surgery as they were over 65 years of age and in a poor GCS score and medical condition, and the family of 2 patients declined surgical intervention. They all died within few days to weeks later from their presentation. Among 6 surgically treated patients, 4 were males (66%) with a median age of 22.5 years and a mean of (40.6±18.5). The most frequent initial presentation was hemiparesis contralateral to the side of the infarction. All patients presented outside the treatment window for thrombolysis. The median GCS after clinical deterioration and just before surgical decompression was 7, and the mean was (7.3±2.4). All patient had at least one CT scan of the brain showing significant edema & midline shift before surgical intervention, and all received the maximal medical therapy to reduce intracranial hypertension. Only one patient developed hemorrhagic transformation on follow-up imaging before the surgical intervention. The mean time from admission to surgery was (49.2±49.9) hours, with a median of 35.5 hours. The immediate post-operative mean GCS was (6.17±5.2), with a median of 6.5. Two patient developed postoperative complications, in the form of hemorrhagic transformation, within seven days after the decompression. Early mortality measured at thirty-days post-DC was 50%, this rate was unchanged at one-year follow-up. The mean 12-month mRS was (5±1.3), and the median was 4.7. The mean BI was (12.5±24) at 12-months follow-up, with a median of 7.5.

**Table 1:** Summary of cases with malignant middle cerebral artery (MCA) stroke treated with decompressive hemicraniectomy

Case	Age (yr)/Sex	Risk factors for stroke	Hemisphere involved	Stroke to surgery time (hrs)	Immediate pre-operative GCS	24 hrs post-operative GCS	mRS at 12-months follow-up	BI at 12-months follow-up
**1**	48/F	DM, portal vein thrombosis	Right	120	11	15	3	60
**2**	54/M	DM & HTN	Right	3	4	3	6	0
**3**	6/M	sickle cell anemia	Right	64	7	10	4	15
**4**	39/M	HTN, DM, ischemic heart disease	Left	7	7	3	5	0
**5**	40/F	HTN	Left	91	9	3	6	0
**6**	57/M	DM, HTN, ischemic heart disease	Left	10	6	3	6	0

HTN - Hypertension, DM - diabetis millietus, GCS - Glasgow Coma Score

## Discussion

There is a consensus that decompressive hemicraniectomy decreases the rates of mortality among patients with malignant hemispheric strokes.^3^,^5^,^6^,^16^,^18^-^26^ Despite the clear advantages of decompressive hemicraniectomy in these patients, several questions and controversies remain, including which patients are suitable surgical candidates and whether death is merely being replaced by survival with major disability.^10^ There is no strong evidence evaluating patient-related factors such as; co-morbid conditions, baseline and pre-operative GCS, and the timeline for clinical deterioration, hence identifying those who will benefit from surgical decompression. The DECIMAL trial made attempt to pinpoint predictors of response, identifying moderate direct correlations between infarct volume on diffusion-weighted MRI and 6-month mRS (r=0.53, *p*=003) for the 38 patients enrolled, with a weak correlation between patients’ age and 6-month mRS (r=0.64, *p*=0.002). No correlation was observed in the non-surgically treated patients.^4^ Hao et al^15^ prospectively assessed 2174 patients with acute ischemic stroke seen at Sichuan University Hospital between December 2007 and March 2011, of whom 219 (10.1%) were ultimately diagnosed with a malignant MCA infarction and 31 of these 219 (14.2%) underwent decompressive surgery. Outcomes were assessed at both one-month and one-year post-stroke, with survival at these time points equal to 67.7 and 61.3% in those treated surgically (n=31), respectively, versus 48.9 and 38.8% in their non-surgical counterparts (n=188). The proportion of patients achieving a mRS ≤3 were 32.3 and 11.7% (OR=3.59; 1.50, 8.62; *p*=0.006), while those with a one-year mRS ≤4 were 51.6 versus 25.0% (3.20; 1.47, 6.97; *p*=0.002). Although this was not reported, further analysis of the reported numbers shows that, among survivors, only 15.8% were totally dependent (mRS=5) versus 33.8% of their counterparts, a difference that fails to achieve statistical significance (OR=2.72; 0.72, 10.27; *p*=0.14). However, this demonstrates that the increased survival observed with surgery did not yield a greater percentage of patients who were totally dependent for all needs, a concern that has been raised by others.^15^ On multivariate analysis, the severity of the initial stroke (as rated using the NIHSS instrument) and surgery *p*=0.016 versus no surgery *p*=0.004 were the 2 remaining predictors of survival at both one month *p*=0.009 and one year *p*=0.012, while surgery *p*=0.015 and patient age ≤ 60 *p*=0.012 predicted a good outcome (mRS≤3) at one year. Comparing survival in those ≤60 versus >60 years old rates were 73.9 versus 50.0%, one-month and one-year survival rates were 69.6 versus 37.5%, but these differences failed to achieve statistical significance. Similarly, those who underwent surgery within 48 versus >48 hours since their stroke had enhanced survival at both one month and one year (71.4 versus 52.9% and 71.4 versus 47.1%), but these differences again were not statistically significant.

Evidence from randomized and non-randomized studies have also supported improved outcome with decompressive hemicraniectomy in older patients. In a randomized trial of 112 patients with malignant MCA syndrome who were older than 61 years (median of 70 years), survival without severe disability at 6 months from randomization was significantly higher in the decompressive hemicraniectomy arm (38%) compared to the conservative arm (18%; OR 2.9, *p*=0.04).^27^ In a non-randomized study in India, 79 consecutively admitted patients with malignant MCA stroke with 37 undergoing decompressive hemicraniectomies. Survival rates at 6 months in the surgery versus non-surgery groups were 81.1% and 28.6%, which represents more than a 50% absolute reduction in mortality.^28^ This enhanced survival was also evident in patients over age 60, with 83.3% of the 24 older surgical patients alive at 6 months versus just 26.3% of their 38 non-surgical counterparts (*p*<0.001). Predictive of survival were surgery and the patient’s pre-operative GCS and Acute Physiology and Chronic Health Evaluation II (APACHEII) score. On receiver operating characteristic (ROC) analysis, a pre-operative GCS ≥ 8 and APACHE II score≥13 were the thresholds that best combined sensitivity and specificity for survival, with sensitivity and specificity values of 100 and 84.4% for the GCS (*p*=0.003) and 80.0 and 96.9 for the APACHE II rating (*p*=0.009). The absence of hypertension also predicted survival in patients over age 60 years. Hypertension also was found to predict mortality in an Italian study by Caso et al^29^ in which 125 patients with MCA strokes were analyzed 28. Of this number, 44 (35.2%) died in hospital. On univariate analysis, median diastolic blood pressure (DBP) at admission was 90 mmHg in patients who died versus just 80 mmHg in survivors (*p*=0.01). Moreover, rates of mortality were 22, 56 and 67%, respectively, in patients with DBP below 90 mmHg, from 90 to 109 mmHg, and 110 mmHg or above. Overall, after adjusting for other risk factors, the risk of death increased by 5% for each one mmHg increase in admission DBP. On multivariate analysis, both an elevated admission DBP (OR=1.05; 1.01, 1.09) and high National Institutes of Health Stroke Scale (NIHSS) score (OR=1.17; 1.03, 1.34) were independent predictors of in-hospital mortality.

We operated on 6 out of 10 patients who presented with malignant hemispheric stroke, and hence these numbers do not allow for any meaningful statistical comparisons between survivors and non-survivors. Nonetheless, several findings are of note. First, survival percentage was similar to another series of four patients reported by Swiat et al^30^ in 2010, in which 2 patients died, and 2 had residual moderate to severe disability (mRS=4) at 12-month post-operative follow-up.

Second, all our patients were under age 60 as we declined to operate on the 2 older patients who were in poor health as a baseline. Differences encountered between the survivors, and the non-survivors were (1) non-survivors experienced their post-admission clinical/neurological deterioration necessitating surgery over a matter of a few hours (both within 5 hours) versus 22 and 24 hours in survivors (t=18.00, *p*=0.003); and (2) that the absolute drops in GCS were greater and the final GCS significantly lower in the 4 the non-survivor (GCS=3) compared to the survivors (GCS=15 and 7).

In our series, the surgical mortality rate was equal to 50%, the highest among the reported in the literature,^31^-^32^ which can be explained by the small size sample. The overall complication rate was relatively low (33%) this number is lower than the recently published numbers reported by Kurland (47%).^33^ Furthermore, we found no significant improvement encountered in the functional independence with mean BI 12.5±24, which was less favorable compared to the numbers reported by Fandino et.al in 2004 (47±25).^34^ We also found no significant effect on the reduction of moderate-sever disability (mRS >4=66%). We accept the numerous limitations of our study, beginning with the very small number of surgical cases, which prevented any analysis of potential confounders. It may be that certain finding on imaging significantly influence outcomes, for example, the volume of ischemia on diffusion-weighted images or some objective measure of the extent of midline shift which was not evaluated in the current report. Our findings warrant consideration in future research to identify predictors of survival and functional outcomes in patients undergoing decompressive hemicraniectomy for malignant hemispheric (MCA) stroke.

In conclusions, the factors predicting patient’s outcome following decompressive hemicraniectomy should receive more attention to specifying patients who are likely to benefit from the decompression. Once such subset of patients is identified, early surgery should be offered within the first 24 hours before clinical deterioration occurs, and early referral of such cases to the neurosurgeon is advised. The variations in the surgeon’s knowledge and attitude towards decompressive hemicraniectomy could be a major factor and should be studied thoroughly.
